# The Terminal Segment of the Seminiferous Tubule: The Current Discovery of Its Morphofunctional Importance in Mammals

**DOI:** 10.3390/cells14040305

**Published:** 2025-02-18

**Authors:** Vicente Seco-Rovira, Ester Beltrán-Frutos, Jesús Martínez-Hernández, Juan Francisco Madrid, Luis Miguel Pastor

**Affiliations:** Department of Cell Biology and Histology, Medical School, IMIB-Arrixaca, Regional Campus of International Excellence “Campus Mare Nostrum”, University of Murcia, 30120 Murcia, Spain; vicente.seco@um.es (V.S.-R.); ebf96527@um.es (E.B.-F.); jesus.martinez7@um.es (J.M.-H.); jfmadrid@um.es (J.F.M.)

**Keywords:** testes, terminal segment, proliferation, sertoli cell, seminiferous epithelium

## Abstract

The morphophysiology of intratesticular sperm pathways in mammals, including humans, is poorly understood. The seminiferous tubule is continuous with the straight tubule; however, its final portion—the terminal segment (TS)—has a different tissue composition. This paper reviews the most important histological results from mammal studies from the last decades of the 20th century, including the different nomenclatures given to the TS. The TS presents a loss of spermatogenesis and is lined mainly with modified Sertoli cells. There is no unanimity among authors when it comes to naming and defining TS. In the last ten years, studies on rats and mice have highlighted the importance of this testicular zone, proposing that there is a high proliferation of modified Sertoli cells with an undifferentiated cellular profile associated with stem spermatogonia. In hamsters, an immunohistochemical study showed the existence of heterogeneity between these cells, and the surrounding interstitium presents numerous Leydig cells that are ultrastructurally different from those of the rest of the testis rest. In conclusion, we have only just begun to understand the tissue biology of TS. Emerging research is very promising; it can potentially modify our current knowledge of testicular biology and be very useful in promoting the advancement of male fertility restoration therapies in andrology.

## 1. Introduction

Mammalian intratesticular excretory pathways are defined as the region from the end of the seminiferous tubules to the rete testis (RT) and are commonly divided into the terminal segment (TS) of the seminiferous tubule, the straight (recti) tubule (ST), and the RT. The TS has recently been characterized as having cells with a high differentiation potential [1,2]. The TS has been the subject of past research; however, we lack a clear understanding of its components. Current studies are not in agreement regarding the cell populations present or the phenotypic variations between them. In this work, we first performed a bibliographic review of the current literature in this area, summarizing and systematizing the findings of the last 60 years, while also affording new data revealed in our latest studies on the TS in Syrian hamster testes, an animal model widely used in reproductive biology studies.

## 2. Results

### 2.1. The Terminal Segment of the Seminiferous Tubule and Its Tissular Structure in Mammals

#### 2.1.1. Light Microscopy Results

In the 20th century, the first studies emerged that aimed to define and describe the TS location histologically. In 1964, Marin-Padilla [3] described this zone as the final portion of the seminiferous tubule formed by Sertoli cells (SCs) invaginated into a dilation of the ST (“receptacle”). This establishes communication between the gonads and the mesonephric excretory system during sexual maturation in humans and other mammalians, such as horses, armadillos, and hamsters. This author considered that the “receptacle” was a structure that, together with the ST and RT, would constitute a specific organ: the rete organ. On the other hand, other authors considered that the final part of the seminiferous tubule, which they called the ST, contains only SCs, considering the “receptacle” as a mere physical dilation of the ST in the mouse [4,5]. In the hamster, the TS is described as comprising SCs, constituting a final plug. This segment is shorter than that observed in the guinea pig or monkey [6]. Also, at that time, other authors called this location the “intermediate region” [7,8]. Perey et al. (1961) [7] revealed that in a rat, the union between the seminiferous tubule and the RT is produced through a funnel-shaped tube composed of two parts: one that opens toward the RT and the intermediate duct (only with SCs), which is continuous with the seminiferous tubule provided with its characteristic epithelium. The intermediate duct has an epithelium of tall cells with a nucleus characteristic of SCs. The apical extensions of these cells form a papilla that projects toward the ST and possibly functions as a valve that allows liquids and free cells to exit—but not enter—the tubule. However, in guinea pigs, Vitale-Calpe and Aoki (1969) [8] reported that they did not find the plugs in the intermediate region, as described by other authors. Instead, they considered this region to be lined with modified Sertoli-type cells, providing the first ultrastructural description of these cells.

#### 2.1.2. Electron Microscopy Results

As early as the 1970s, Dym (1974) [9] introduced the term “transitional zone” for this location. In his light and transmission electron microscopy (TEM) study in monkeys, he considered that there is a gradual depletion of the most mature germinal elements in the final part of the seminiferous tubule until the tubule is lined only with SCs and spermatogonia. Then, the spermatogonia disappears, and the TS of the tubule is lined only with SCs. In the adult macaque, the terminal portion, synonymous with the transitional zone, is characterized by unusual SCs with a high presence of cytoplasmic filaments. In addition, degenerating spermatozoa are found within the cytoplasm of some of these cells, as well as some lymphocytes between the SCs. He considered that the epithelium of the terminal portion of the seminiferous tubule was mainly composed of SCs, which sometimes seemed to block the lumen. However, they thought this appearance was attributable to oblique or tangential cutting planes, giving the erroneous impression of a “plug” occluding the lumen. In the guinea pig, Fawcett and Dym (1974) [10] also used the term “transitional region” of the seminiferous tubule to refer to the “intermediate region” of Perey et al. (1961) [7]. Nevertheless, they also described this location using a more integrative term: “terminal segment of the seminiferous tubule”. For them, the cytoplasmic prolongations of the SCs project a certain distance into the lumen of the ST near its junction with this region. The authors emphasized the presence of direct communication with the ST, as a central mass composed of apical portions of SCs, separated by a narrow cleft with a surrounding layer of low cuboidal cells, is occasionally observed in transverse or oblique sections through this junction region. This appearance would be responsible for the earlier descriptions of a “plug”-shaped mass of SCs. They concluded that, when sectioned favorably, there is always a small central lumen throughout the final region of the seminiferous tubule.

Between 1978 and 1988, some ultrastructural studies were performed on several mammalian species. In 1978, Osman [11] summarized the definitions of this zone and reused the term terminal segment (TS) of the seminiferous tubule, referring to the part anterior to the ST in the pig testis. He defined the seminiferous tubules as being attached to the ST by a short TS, which is lined with a single layer of modified SCs. These cells have long cytoplasmic processes that occlude the lumen, forming a plug-like structure in the “receptacle”. In this study, he could only observe traces of the central lumen, concluding that the long cellular processes, loosely arranged in the “plug”, can easily collapse. Nevertheless, due to fluid pressure flow, these processes would maintain a permanent communication between the seminiferous tubule and the ST. Furthermore, supporting this hypothesis in the rabbit and rat, he found that this lumen was clearly observed when under increased intratesticular pressure after ligation of the efferent ducts [12,13].

This same author found that this segment is lined with modified SCs with abundant microtubules, microfilaments, crystalloids, and rough endoplasmic reticulum in the cytoplasm. He also observed dilated intercellular spaces and that the cells have wide intracellular spaces in the cytoplasm; the cells were joined by desmosomes and tight junctions. In addition, he observed degenerated spermatozoa engulfed by these modified SCs. Following the nomenclature of TS for the final zone of the seminiferous tubule, Osman, in 1978 [14], characterized the ultrastructure of epithelial cells at this location in three different species: bull, ram, and goat. This study confirmed, in these species, that the segment is lined with modified SCs, characterized by intercellular dilatations and abundant microtubules and microfilaments in their cytoplasm. The presence of vacuolated nucleoli and typical junctions between SCs confirms that these cells are modified SCs since they possess special characteristics compared with normal SCs.

In 1979, Nykänen [15] analyzed the tissular structure of the transition zone of the seminiferous tube in the rat. Following the nomenclature of Dym (1974) [9] regarding this location, he also described it as being lined with modified SCs, with only a few spermatogonia and macrophages, and with a very narrow lumen distally. This author observed an increase in microtubules in the columnar portions of the cells. Lipid droplets and lysosomal structures are also frequent cellular components of SCs, as are empty intracellular vacuoles that are sometimes arranged around areas of smooth endoplasmic reticulum. The apical processes of SCs display large vacuolar structures, and capillary-like vacuoles are frequently seen in the basal parts of the epithelium. Phagocytosis of germ cells by SCs occurs in the proximal region of the transitional zone. Rounded debris bodies in contact with the apices of SCs that protrude into the ST are also common. The tunica propria of the entire zone is thickened and somewhat wrinkled. Junctional complexes between SCs appear impermeable to the lanthanum tracer, indicating an intact blood–testis barrier.

In 1982, Lindner published the first human study that combined optical microscopy and TEM of this testicular portion [16]. Regarding the nomenclature, the term “transitional zone”, coined by Dym (1979] [9], was adopted and combined with the concept of the TS. In this study, the presence of some confusion in the nomenclature was pointed out, and it was considered that, following Osman (1978) [11], the term TS would be used to refer to the final part of the seminiferous tubule that connects it with the ST. This author described the lumen of the TS as narrow and presenting a lamina propria with a considerable thickening with respect to the normal one of the seminiferous tubules. At the end of the TS, as in other species, the modified SCs were seen to project toward the lumen of the ST, forming a structure described as a lip. Compared with the SCs of the seminiferous tubule, those of the TS are described with specific modifications: nuclei are oval and do not present clefts in the membrane. Also, there are no perinuclear cytoplasmic inclusions and there are numerous lipid droplets and residual bodies in the apical region of the cells. Furthermore, a greater number of microfilaments and microtubules were observed throughout the cytoplasm. The lamina propria consists of a basal lamina (BL) and myofibroblasts, which are arranged in an irregular structural pattern. Between these myofibroblasts, there are thick bundles of collagen fibrils that surround the TS, constituting a ring. At the same time, Wrobel (1982) [17] published a description of the TS in the bull using TEM. They distinguished three portions: the transitional region, the intermediate portion, and the terminal plug, the latter two being formed almost exclusively by modified SCs. Wrobel was the first to divide the TS into three zones, affording a different meaning to the transitional term, as it does not refer to the portion with SCs or modified SCs but rather to the previous part of the seminiferous tubule, where some germ cells remain. He also considered that the cells in the intermediate portion and terminal plug are not identical to the SCs of the seminiferous tubules. In addition, each TS is surrounded by a vascular plexus. The modified supporting cells of the midportion and terminal plug no longer exhibit the typical Sertoli-Sertoli junctions seen in the transition region and seminiferous tubule. A distinct central lumen is generally not seen in the terminal plug region; sperm and tubular fluid must pass through an intricate system of communicating slits between the apices of the tightly packed modified supporting cells. Vacuoles in the supranuclear region of midportion cells indicate that there must be a strong transepithelial fluid transport. Analogous to the RT and ST epithelium, the supporting cells of the TS can phagocytose sperm. The vascular plexus lining the TS would, according to this study, fulfill a dual function: a regulatory device for fluid and sperm transport, as well as an area of increased diapedesis for white blood cells. In this study, and after ultrastructural analysis, it was not possible to classify different types of supporting cells in the TS. The differences were only quantitative, such as the number of filaments, microtubules, or free ribosomes in the cytoplasm. In this species, a variable number of phagocytosed spermatozoa in different stages of degradation was also observed in the cytoplasm of epithelial cells. In the interstitium, some Leydig cells immediately adjacent to the TS presented a less-developed functional differentiation than Leydig cells in the center of the testicular parenchyma. The Leydig cells were smaller and often had a spindle shape. The study concluded that, in the bull, there seems to be a terminal plug, which, under normal conditions, functions as a valve-like device preventing the reflux of spermatozoa and fluid from the ST.

The last major ultrastructural studies were performed on camels and pygmy goats in 1986 and rats in 1988. In pygmy goats [18], modified SCs constitute the TS epithelium. They resemble standard SCs but differ in the paucity of agranular endoplasmic reticulum and lipid droplets. Cell attachment mechanisms include rudimentary desmosomes and occasional multiple contacts of opposing plasma membranes, interrupted by segments of slightly expanded intercellular space. There are a few germ cells in the initial zone of this epithelium. The epithelium of the middle zone of the TS comprises vacuolated cells that lie between other cells containing abundant microtubules in their subapical cytoplasm. Globular expansions of the intercellular space are also evident. In the final part, the terminal plug contains two types of cells. Type I are slanted, columnar cells containing abundant agranular endoplasmic reticulum formations in their apical cytoplasm. Type II cells are smaller and located at the apex of the plug. Modified SCs from all zones and Type II plug cells contain sperm remnants in various stages of degradation, indicating spermatophagy activity.

Also, in 1986, Osman [19] published an article on the intratesticular excurrent ducts of the camel, which included a characterization of the TS of the seminiferous tubule. This was lined with modified SCs that formed a plug-like structure in the receptacle. Many spermatozoa were found inside the modified SCs of the TS. This author insisted that this region is known as the TS of seminiferous tubules and that the columnar cells of its epithelium are modified SCs. In addition, a central lumen was seen in the TS in most of the sections examined; the distal parts of the modified SCs formed a plug-like structure inside the receptacle.

Hermo and Dworkin (1988) [20] performed a detailed ultrastructural study of modified SCs from this region in the rat, considering their cytoplasm, endocytic activity, and basement membrane. Transitional cells, as they called them, line the “intermediate” region of rat seminiferous tubules between the RT and seminiferous epithelium proper. These tall, elongated cells converge on each other distally into the lumen, forming a papilla-like structure through which a narrow, patent lumen is visible. In addition to a widely dispersed Golgi apparatus and mitochondria, these cells contain abundant microtubules, endoplasmic reticulum cisternae, and a lobed nucleus displaying a prominent nucleolus. According to these results, these cells would actively participate in both endocytosis and adsorption of tubular fluid, playing an important role in modifying its composition. In particular, transitional cells of the distal zone of the intermediate region rest on a complex BL. This includes a first thin BL and, immediately below, a thick BL layer with BL strands forming an anastomosing network. The use of antiserums against heparan sulfate proteoglycan or the proteins laminin and type IV collagen revealed the presence of these three components in all areas of the complex BL. They observed extracellular vesicles between the meshes of the BL network. They also found vesicles of similar size and appearance in the basal region of the epithelial cells and in their basal feet, which extend deep within the meshwork of the BL network.

### 2.2. Assessment of Terminal Segment Studies in the 20th Century

In summary, the initial histological studies conducted on the TS across various mammalian species revealed that the epithelium of seminiferous tubule consistently transitions at its terminal end into a segment composed solely of modified Sertoli cells (SCs), along with some spermatogonia in its most proximal region. This segment exhibits remarkable similarity across all species examined and is initially referred to as the intermediate segment in rats and guinea pigs [7,8]. It is later termed the transitional zone in monkeys, rats, and humans [9,15,16] and, subsequently, identified as the terminal segment (TS) in hamsters, boars, and bulls [6,11,17]. In the distal portion of this tubular region, modified SCs form a valve or plug-like structure that connects seamlessly with the ST, which includes an initial section known as the receptacle, leading into the RT. Some researchers have mistakenly interpreted this description, suggesting that the TS represents the first part of the ST based on early observations in mice and rats [4,5]. This interpretation is inaccurate, as the ST is the segment that links the TS to the RT [6,10]. In addition, ultrastructural evidence indicates that phagocytosis and intracytoplasmic degradation of spermatozoa occur in the modified SCs. Various studies in diverse species have observed the presence of an initial zone of the TS in which the spermatogenesis process gradually decreases until there is no sperm production, followed by an intermediate zone with modified SCs, and a final zone in which the plug is formed. These observations have given rise to problems in the naming and nomenclature of this location. Initially, the TS was the end of the seminiferous tubule that connected to the ST and was lined only with SCs or modified SCs. However, it was later observed that there was a preceding zone with progressive loss of spermatogenesis. This could be interpreted in two ways: as the last part of the seminiferous tubule or the initial part of the TS; this gave rise to terminological confusion. Thus, if we follow the first option, the TS would be everything that comes after the seminiferous tubule with modified SCs; with the second option, modified SCs would be in a transitional or intermediate region, which would form the valve or plug in its most distal part. Likewise, to confuse things even further, some authors used the word “transitional” to name the portion of the TS with gradually disappearing spermatogenesis, which would be followed by the intermediate and then the terminal zone in the form of a plug. Even with these difficulties, the tissue structure of this final part of the seminiferous tubule can be clearly characterized. The conclusions derived from the conducted studies reveal that in mammals the seminiferous tubules connect with the ST through its final part, the TS. This comprises a proximal subpart in which the epithelium progressively loses spermatogenesis with its corresponding SCs, followed by another subpart with only modified SCs, and then a final or distal part connecting to the ST, where modified SCs are configured to form a plug. This plug seems to exhibit a narrow lumen that changes according to the pressure exerted by the liquid in the lumen of the tube. Finally, it is noteworthy that several authors studying the TS at the tissue level have reported significant modifications in both its tubular wall (peritubular interstitium) and the underlying intertubular interstitium. These are either due to the presence of an important vascular plexus, cells of the immune system, or the existence of important groups of Leydig cells, some of them in the process of differentiation.

### 2.3. Recent Interest in Experimental Andrology at This Location

In addition to its potential role as a valve regulating spermatozoa passage and preventing fluid reflux into the seminiferous tubule, two recent discoveries have highlighted the significance of the TS in testicular biology. The first is the possibility that this area may be a “niche” for trunk spermatogonia; the second is the possible existence of SCs with a phenotype different from the SCs normally found in seminiferous tubules with complete spermatogenesis. This phenotype would be related to the fact that these cells have not lost their proliferative capacity and present a lower state of differentiation. These observations suggest that in this ST, SCs may possess specific properties that would help maintain gonadal stem cells and that their proliferative capacity serves to maintain the balance of the SC population in the seminiferous epithelium [2,21].

#### 2.3.1. SC Proliferation in the Adult Seminiferous Tubule

The SC in the seminiferous epithelium plays a key role in spermatogenesis, acting as a support for other germ cells. It performs a multitude of functions; however, the most important are those of a structural and functional nature with respect to the other epithelium cells. SCs provide them with nutrients or necessary external signals, e.g., growth factors to maintain spermatogenesis [22], in addition to participating in the regulation of germ cell differentiation [23]. The importance of SCs in spermatogenesis is such that the daily number of male gametes produced depends directly on the number of SCs in the testis [24]. The final SC number is reached once puberty ends [25,26], and to do so, SCs divide at certain times in the life of an individual, such as during the fetal period and puberty, when there is an increase in the total SC number due to their proliferation [26]. There are hormones known to stimulate SC cell division and others that promote their differentiation to the adult SC state. Thus, on the one hand, follicle-stimulating hormone (FSH) promotes SC proliferation, while testosterone accelerates its differentiation [26,27,28]. Traditionally, it was considered that once adulthood was reached, the SC was fully differentiated and, consequently, quiescent. This implies that its total number is stable and invariable during an individual’s lifespan, not responding to the hormone that previously stimulated its division [26,29,30]. These ideas are currently under discussion. Years ago, some authors suggested that the SCs of adult individuals could modify their mature, differentiated state to a more immature and undifferentiated state under the effect of gonadotropins. This indicates the existence of an SC population in a dynamic, hormone-modifiable state, at least in seasonal breeders such as the Russian hamster [28]. This hypothesis was supported by other studies that, using stereological techniques, had previously described variations in SC number in adult Russian hamsters [31,32,33] during their regression and testicular recrudescence. Furthermore, in parallel, recent in vitro studies have described that SC division implies that they express all the “proliferative machinery” necessary to this end [34,35]. Furthermore, in the Syrian hamster, the proliferation of adult SCs has been observed both under long photoperiod conditions and during testicular regression due to short photoperiods. Indeed, during testis recrudescence following its reversible regression due to short photoperiods, SC proliferation significantly increases until the seminiferous epithelium is recovered [36].

#### 2.3.2. SC Proliferation and Cell Dedifferentiation of Modified Sertoli Cells in the Terminal Segment

As mentioned, several authors described SC proliferation in the adult TS under normal physiological conditions. In the Wistar rat, SC proliferation was observed between the seminiferous tubule and the RT, which only contains SCs and some spermatogonia. In addition, it was suggested that there is a subpopulation of these undifferentiated cells at this location. From there, the seminiferous tubule, thanks to this SC population, could grow when necessary [1,21], although there is still no clear demonstration of this function in the adult rat. These proliferating SCs were found only in the TS of both prepubertal and adult rats. Ki-67 analysis on day 36 revealed that approximately 4% of SCs in prepubertal rats were proliferating in the TS. This activity was significantly reduced (less than 1%, *p* < 0.05) in adult rats. p27 immunostaining exhibited a similar negative pattern, with p27-negative SCs found exclusively in the TS in both the prepubertal and adult stages in similar proportions to proliferating SCs. A similar occurrence was observed with the transcription factor GATA-4. Approximately 8% of SCs in the TS were GATA-4 negative in prepubertal rats, whereas in adults, this figure was significantly reduced (4%, *p* < 0.05). Very few GATA-4-negative SCs (less than 1%) were observed in young and adult rats. Androgen receptor (AR) expression in SCs exhibited a pattern consistent with the above. AR-negative SCs were frequently observed in the TS of prepubertal rats, as 17% of SCs did not express ARs; in adult rats, this non-expression was reduced to about 8% (*p* < 0.05). A later study [1] summarized the TS tissue profile, which was positive for cyclin D-1 and FGFR2, or weakly positive for differentiation markers, such as DMRT1. A decrease in connexin 43 expression and strong expression of S-100-alpha or GNDF in most modified SCs was also reported. All these results indicate the existence of a different SC population with varying degrees of differentiation at this location in the rat. Another species in which the TS has been studied is the mouse. The phosphorylated signal transducer and activator of transcription 3 (p-STAT3) were predominantly observed in SCs of the valve adjacent to the RT [37]. In the distal seminiferous tubules with active spermatogenesis, most SCs were negative for anti-p-STAT3 immunostaining, which was positively correlated with GDNF expression. These facts could be related to the possible niche function of the TS for spermatogonial stem cells. More recently, in mice, Imura-Kishi et al. (2021) [38] confirmed the importance of the TS as this spermatogonial stem cell niche. SCs in the TS express the Cyp26a1 gene, which controls retinoic acid degradation, thus maintaining lower levels of retinoic acid in this segment [38]. Retinoic acid is essential for the differentiation of undifferentiated spermatogonia A [39,40]; therefore, maintaining reduced levels in the TS would ensure the maintenance of a spermatogonial stem cell niche. Furthermore, Imura-Kishi et al. (2021) [38] found fewer undifferentiated GFRα1-positive spermatogonia A after retinoic acid microspheres were transplanted into the TS. This demonstrated that the TS could retain spermatogonia in an undifferentiated state until required under normal conditions in the adult testis. Recently, the transcription factor Sox17 has also been found to be expressed in the RT epithelium [2]. In Sox17-knockout mice, the TS was disrupted before puberty, which induced reflux of RT fluid into the seminiferous tubule, causing an aberrant shedding of immature spermatids. The TS of Sox17-cKO mice had reduced expression levels of several growth factor genes, presumably aiding TS formation. When transplanted into the TS, Sox17+ RT cells and Sox17-cKO SC mice rebuilt the TS and helped recover spermiogenesis. This study demonstrated that the TS has modulatory functions in spermatogenesis in mouse testes. In summary, the expression of Sox17 modulates the TS, demonstrating that it is essential for its formation and spermiogenesis in the seminiferous tubule.

Other works also support that the TS is involved in the active maintenance of the seminiferous epithelium; thus, cells at this location are sensitive to thyroid hormones. When Wistar rats are subjected to transient hypothyroidism during postnatal development, delaying testicular maturation, a significant increase in testicular weight, SC number, and daily sperm production is obtained. This result suggests that these SCs exhibit plasticity and can be stimulated to increase their proliferation, contributing to a late increase in testicular weight and sperm production [1,41]. This would imply a physiological role of the TS in the pubertal maturation of the testis toward the adult state. Similarly, in the Syrian hamster, one year before Figueiredo’s studies (2016) [21], a “niche” of proliferating SCs had already been described in the same area in adult individuals and under normal physiological conditions [42]. This was associated with the presence of undifferentiated spermatogonia, suggesting that this region could be a “niche” for these, which would participate in their replacement in the seminiferous epithelium. Another study in the mouse and using cell cultures determined the existence of two SC populations in adults. The first population arose from the seminiferous tubules of testicular parenchyma and barely proliferates in vitro. The second is small and comprises ST cells that proliferate in culture and form colonies, displaying a mixture of the characteristics of mature and immature SCs [35]. Finally, in recent years, some authors have drawn attention to the fact that some lymphocytes could be found beyond the BL of the TS, ST, and RT. These are located close to the testicular spermatozoa of the TS lumen. These findings in mice raise the possibility that lymphocytes may be in contact with sperm autoantigens under physiological conditions [43]. Furthermore, some macrophages were reported to have penetrated the epithelium, and the density of F4/80-positive macrophages was significantly higher in the area around the TS than the RT [44]. In addition, lymphocytic infiltration can initiate around the TS under various pathological conditions [43,45]. During experimental autoimmune orchitis [44], lymphocytes preferentially accumulate in the TS, secreting several cytokines, interferon-γ, and tumor necrosis factor-α. In summary, current evidence suggests that this region may be closely involved in the onset of inflammatory pathologies. Furthermore, the BL of the TS has also received increased attention in recent years due to its differences from that of the seminiferous tubule. A comparative ultrastructural study showed that the BL of modified SCs from the TS has a wavy and multilayered structure; however, the epithelium of the RT and the seminiferous tubule has an almost flat and single-layered structure. This characteristic structure of the BL of the TS could be related to its valve function and potential interaction with lymphocytes that recognize autoantigens [46].

**Figure 1:**
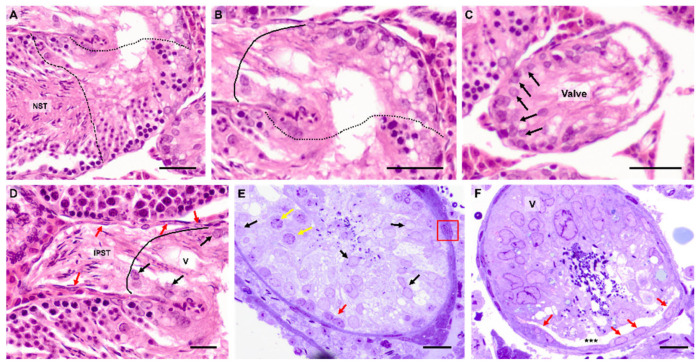
Light microscopy. Terminal segment of the hamster seminiferous tubule. (**A**) Seminiferous tubule with normal spermatogenesis (NST), followed below by the first subpart (between the dotted lines) in which the epithelium progressively loses spermatogenesis; hematoxylin and eosin staining (H&E). (**B**) Subpart with only modified SCs and a final zone of modified SCs that form a plug or valve (between continuous and dotted lines); H&E. (**C**) Modified SCs (arrows) in the plug or valve; H&E. (**D**) Receptacle or the initial part of the straight tubule (IPST). The continuous line demarcates the end of the SC valve (SV) with modified SCs (black arrows). The IPST exhibits a flattened epithelium (arrows red). Valve (V); H&E. (**E**,**F**) Semithin sections stained with Toluidine blue. (**E**) Some spermatogonia (red arrow) and spermatocytes (yellow arrows) are found in the TS epithelium. SCs: black arrows. The peritubular interstitium shows several layers of myoid cells (red square). (**F**) Clearly shows the valve plug (V). Flatted cells of the IPST epithelium (red arrows) and IPST lumen (asterisks). Scale bar = 25 µm. From [47,48].

**Figure 2:**
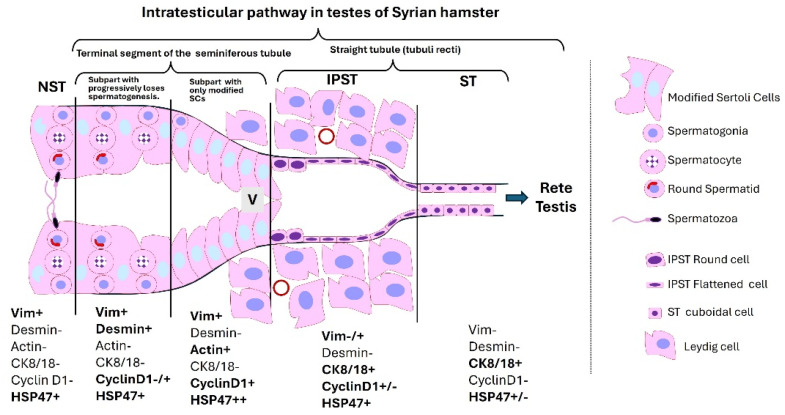
Summary of the results obtained in the terminal segment epithelium of Syrian hamster seminiferous tubules. NST: Seminiferous tubule with normal spermatogenesis. IPST: Initial part of the straight tubule. ST: Medial and final segment of the straight tubule. Vim: Vimentin intermediate filaments. CK8/18: Cytokeratin 8/18 intermediate filaments.

**Figure 3:**
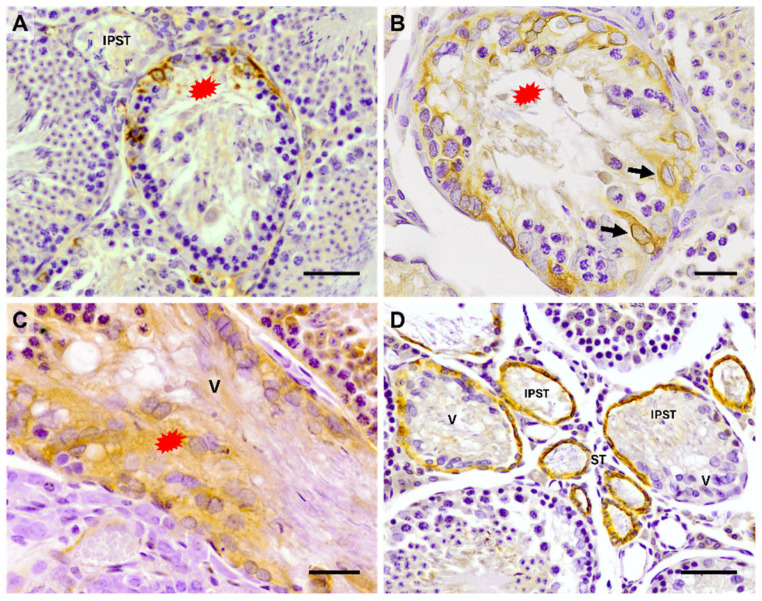
Light immunohistochemistry detection of desmin, actin, and cytokeratin 8/18 in the hamster TS and ST. (**A**) Desmin-positive SCs are only located in the initial subpart of the TS (red asterisk), where the epithelium progressively loses spermatogenesis. In the initial part of the straight tubule (IPST) and seminiferous tubules, in which spermatogenesis is complete, SCs are desmin-negative. (**B**) Transversal section of the initial subpart of the TS (red asterisk). Arrows: desmin-positive SCs. (**C**) The cytoplasm of modified SCs in the valve (V) is highly positive for actin (red asterisk). (**D**) Immunoreactivity to cytokeratin 8/18 is found in the epithelium of the IPST and ST. The modified SCs of the valve (V) are cytokeratin 8/18-negative. Scale bar = 25 µm. From [47,48].

In the hamster—a widely used animal model in reproductive biology—our research group verified some of the aforementioned results (Figure 1) [47,48]. We also obtained new information regarding the modified SCs of the TS by studying other cytoplasmic markers, particularly those linked to the cytoskeleton and cell proliferation (Figure 2 summarizes the results). In the Syrian hamster, we observed varying positivity in the expression of different cytoplasmic filaments, such as vimentin, desmin, and actin (Figure 2 and Figure 3).

**Figure 4:**
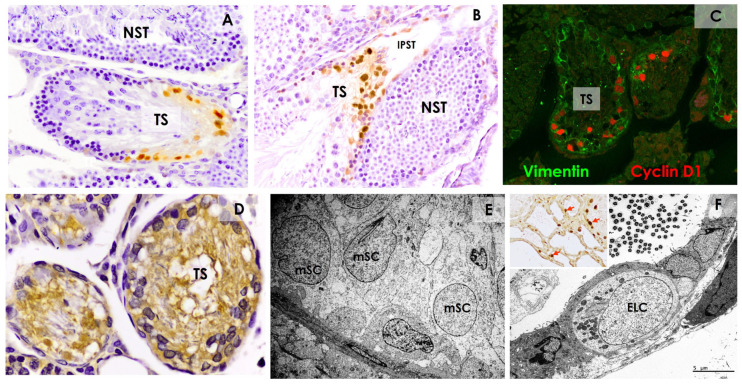
(**A**,**B**) Cyclin D1 immunohistochemistry. Most modified Sertoli cells (SCs) of the TS are Cyclin D1-positive, as are some from the initial part of the straight tubule (IPST). In normal spermatogenesis (NST), positive SCs are not observed. (**C**) In this double-stained confocal microscopy image, D1-positive cells are also vimentin-positive. (**D**) Modified SCs of the TS are strongly positive for HSP47. (**E**) Low-magnification electronography of modified SCs (mSC) with transmission electron microscopy (TEM). Indentations are seen in some nuclei, whereas others are more spherical. (**F**) Electrolucid cells (ELC) are observed with TEM in the ST and RT. Insert: These cells show proliferation and are positive for proliferating cell nuclear antigen (PCNA) (red arrows). Scale bar: (**A**–**C**) = 50 µm; (**D**) = 25 µm; (**F**) (light microscopy) = 50 µm; (**E**,**F**) = 5 µm. From [47,48].

Moreover, all were positive for vimentin and HSP47 protein, which was recently discovered in SCs [49] and is highly expressed at this location (Figure 4). Regarding cell proliferation, the semiquantitative cyclin D1 protein study revealed a significant increase (*p* < 0.05) in the percentage of positive Sertoli-like cells from the final portion of the seminiferous tubule (56.8%) to the valve (73.8%). This expression decreased significantly in the initial part of the ST (31.9%), with no positive cells detected in the ST. These results indicate that there appears to be continuous SC dedifferentiation from the seminiferous tubule toward the transition zone, followed by epithelial differentiation toward the RT. TEM revealed ultrastructural differences between the modified SCs within the TS (Figure 4). Spermatophagy was also observed in the cytoplasm of modified SCs. In the ST and RT, electrolucid cells with traits similar to the modified SCs were observed, along with other cells characteristic of the ST or RT. These isolated electrolucid cells presented proliferative activity (Figure 4).

### 2.4. Leydig Cell Self-Renewal and the Terminal Segment in Adult Testis

The Leydig cell is a very important somatic cell, together with the SC, in testicular function and is responsible for testosterone synthesis [50]. Moreover, the Leydig cell population has been observed to decrease during the lifespan of an individual [51,52]. However, the underlying mechanism and; therefore, how they maintain their population balance and change over time is not well known. Studies on Leydig cell proliferation and death and the relationship between both are scarce. In men, there is a low rate of Leydig cell replacement, and it is suggested that their decrease with aging is not due to dedifferentiation into fibroblasts but to degeneration [53]. Other authors have observed different Leydig cell types with greater or lesser synthetic activity, suggesting that, with age, there could be an increase in the number of cells with low activity—as occurs in various pathological situations—consequently decreasing testosterone production [54]. However, in the hamster, along with this elimination or low functionality, under normal conditions, Leydig cells are replaced to a degree; consequently, there may be different populations of varying ages in the interstitium [55]. This replacement would originate from the proliferation and differentiation of a stem cell, which has yet to be determined. During the first decade of this century, it was suggested that pericytes accompanying the vessels of the testicular interstitium could be the initial progenitors of adult Leydig cells [56] and that Leydig cell death could be due to apoptosis. However, this has always been described under pathological conditions; it is unknown whether this type of death could be important in the normal cellular balance of the interstitium or in the Leydig cell number imbalance that can occur with age [57]. Nevertheless, the age-related decrease in cell number seems to be associated with a reduction in the proliferative activity of pericytes and the constant degeneration of Leydig cells over time [58]. Based on this, a few years ago, we suggested that the loss of Leydig cells with age in hamsters could be related to necrosis. As this necrotic death seems to be a constant phenomenon in different age groups and follows a particular program, we suggested that necroptosis could be the cell death mechanism rather than apoptosis [58]. This hypothesis became more plausible when we found that, during testicular regression due to short photoperiods in this same hamster, a significant loss of Leydig cells occurred—more than 75%—without observing any modification in either the proliferation index or apoptosis index of this cell. We concluded that the reduction in the number during the different regression phases could result from an increase in Leydig cell necrosis/necroptosis [59]. We recently described the expression of a necroptosis marker (RIPK3) in Leydig cells of the Syrian hamster and pig, supporting this possible elimination mechanism [60]. Finally, it should be noted that, since it has long been known that SCs modify both the steroid synthesis of Leydig cells and their proliferation [61], it is necessary to assess the balance of these two cell populations together. Furthermore, as we previously mentioned regarding Leydig cells and their presence around the ST, data are scarce and fragmentary.

In the 1960s, some authors already highlighted the presence of numerous Leydig cells in the interstitium of intratesticular sperm pathways [8]. Cavicchia and Burgos (1977) [6], using optical microscopy in the Syrian hamster, stated that there seemed to be a certain accumulation of Leydig cells in this region of the TS, hypothesizing that it may be a supplementary source of testosterone at this location. More recently, after SC ablation in mice, Leydig cells can only survive at this location close to the TS, thus maintaining testosterone levels in the animals, indicating that these cells probably differ from the other Leydig cells of the testicular interstitium [61].

**Figure 5:**
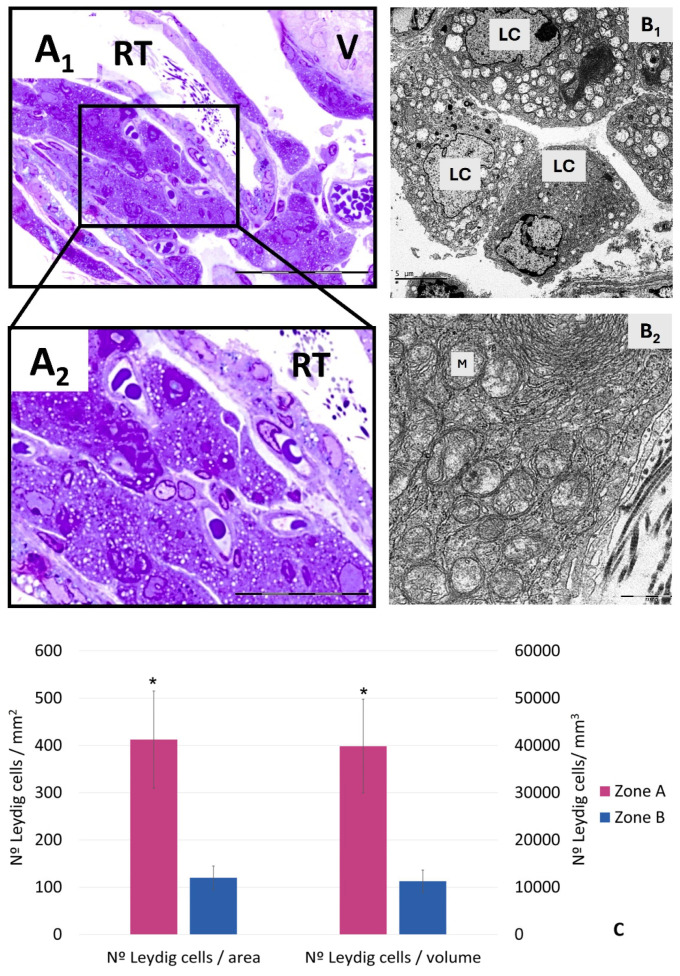
(**A_1_**,**A_2_**) Light microscopy of semithin sections. Hamster intratesticular pathway interstitium. (**A_1_**) shows a large group of Leydig cells can be seen in this interstitium. (**A_2_**) shows them close to blood capillaries. RT: rete testis. V: valve. (**B**_1_,**B_2_**) Transmission electron microscopy. A group of Leydig cells can be seen. LC: Leydig cells. (**B_2_**) is at higher magnification and it is easy to identify large mitochondria (M) as abundant rough and smooth endoplasmic reticula in the cytoplasm. Scale bar: **A_1_** = 50 µm; **A_2_** = 25 µm; **B_1_**,**B_2_** = 5 µm. (**C**) Number of Leydig cells by area (mm^2^) and volume (mm^3^) in zone A (TS) and zone B (remaining testis). Results are expressed as mean ± SD (n = 12 per group). Significantly different results (*p* < 0.05) are expressed as: *—relative to the Zone B group. From [62].

Regarding the hamster intertubular interstitium of this region (Figure 5), we observed different cell types, including fibroblasts, macrophages, and many Leydig cells, which form groups reaching the wall of the first cisterns of the RT. Ultrastructurally, they present abundant mitochondria and a very developed smooth endoplasmic reticulum. Lipid vacuoles and folding of the smooth reticulum are also observed. An increase in Leydig cell density was observed in this interstitium (A), compared with that of the general testis (B), since the number of Leydig cells per interstitial volume was significantly higher in A than in B (*p* < 0.05) (Figure 5) [62]. The initial result found in the intratesticular pathways of the Syrian hamster interstitium is an accumulation or reserve of Leydig cells, which constitutes an important part of their total population in the testis. In addition, these cells present morphological characteristics related to intense functional activity. These results highlight the need for studies evaluating Leydig cell activity in the testis considering these interstitial intratesticular pathways, given their high number and, possibly, their specific properties. As in the case of the SC, exploring the possible existence of a more abundant and probably heterogeneous population of Leydig cells at this location of the TS, as well as its functional role and degree of differentiation, is necessary to determine its involvement in normal and pathological processes of the testis. The TS appears to present a specific and relevant ecosystem that requires detailed study.

## 3. Conclusions and Future Directions

A better understanding of the proliferative activity of modified SCs in the TS, their reversible or irreversible alterations in various physiological and pathological processes, or their degree of differentiation may signify an important change in the direction of fertility studies. This applies to both mammals and humans since, to date, there have been no studies on this subject in the latter in the TS. The somatic epithelial cells of the TS may represent a potential reservoir of undifferentiated epithelial cells, offering promising applications in regenerative medicine and testicular cell therapy. In conclusion, current evidence suggests the possible presence of immature cell populations of the two most important somatic cells of the testis—Sertoli and Leydig cells—at this location. For many years, these cells have been considered quiescent once reaching adulthood, which can no longer be absolutely sustained in mammals. Furthermore, the functional role of this segment during adult life is clearly unknown, as well as how its deterioration due to the passage of time can affect an individual’s fertility. Additionally, it remains unknown how it might behave when faced with harmful agents, endocrine disruptors, various pathologies, or, in the case of seasonal reproduction, in the processes of testicular regression and recrudescence. This region might promote or participate in tubule elongation and preserving the seminiferous epithelium. It might also serve as a site for restoring Sertoli and Leydig cell numbers after testicular injury, making it a potential source of cells for testicular transplants and grafts [1]. Therefore, we can say we are at the dawn of understanding the tissue biology of this area, and improved knowledge may reshape some current perspectives on testicular biology. This also underscores the importance of including this region in future studies to ensure comprehensive results that fully capture the complexity of the testis. Ultimately, more diverse morphophysiological and molecular studies are required to clarify both the role of this TS in normal testicular function, its cellular homeostasis, as well as its participation in various testicular pathologies.

## Data Availability

All data provided have been extracted from the various articles and abstracts consulted.
